# The Canine Frustration Questionnaire—Development of a New Psychometric Tool for Measuring Frustration in Domestic Dogs (*Canis familiaris*)

**DOI:** 10.3389/fvets.2019.00152

**Published:** 2019-05-17

**Authors:** Kevin J. McPeake, Lisa M. Collins, Helen Zulch, Daniel S. Mills

**Affiliations:** ^1^Animal Behaviour Cognition and Welfare Group, School of Life Sciences, University of Lincoln, Lincoln, United Kingdom; ^2^Faculty of Biological Sciences, University of Leeds, Leeds, United Kingdom; ^3^Dogs Trust, London, United Kingdom

**Keywords:** behavior, canine, dog, frustration, psychometric, questionnaire, scale, emotion

## Abstract

**Introduction:** Psychometric tools have been developed for the assessment of behavioral and affective traits in non-human animals. Frustration can be defined as an emotional reaction experienced after a given expectation is violated. Frustration is a negative emotional state and whilst it probably plays a key role in certain behavior problems in dogs (e.g., aggressive behaviors), there appears to have been little attempt to scale this affective tendency. Therefore, the aim of the current study was to develop a tool to assess frustration tendencies in dogs.

**Materials and Methods:** An online owner survey was developed. Items covered demographics, the training/behavioral history of the dog, and 33 frustration related items scored using a 5-point Likert scale. The questionnaire was disseminated via on-line channels over a 5-month period. Two thousand three hundred forty-eight respondents completed the questionnaire. Of these, 273 respondents completed it a second time 6 weeks later, and a separate 276 respondents completed it a second time 1 year later. Additionally, 92 paired responses were collected where two carers completed the questionnaire independently about the same dog. Intra- and inter-rater reliabilities were assessed prior to structuring the items using principal component analysis (PCA) with a Varimax rotation. Items were retained if they loaded > 0.4 on at least one of the components extracted using the Kaiser criterion.

**Results:** Twenty-two items were deemed to be reliable enough to be used in the PCA and 21 items loaded on a biologically meaningful 5-principal component solution. There was a significant positive correlation between each principal component and the owners' general perception of their dogs' frustration tendencies, alongside other expected correlates.

**Conclusion:** This is the first reliable psychometric tool for the assessment of frustration in dogs—the Canine Frustration Questionnaire (CFQ). Further validation with behavioral tests and physiological measures is ongoing.

## Introduction

Frustration has been defined variously as an emotional reaction experienced after a given expectation is violated (1); an animal's reaction following a surprising incentive reduction or omission (2); and as being related to mild engagement of the reactive aggression (RAGE) system which increases in proportion to the intensity of the desire that is thwarted (3). Frustration can arise in a range of circumstances: absent, reduced or delayed rewards (4); situations where one is thwarted from obtaining/retaining a resource (5, 6); where barriers to autonomous control exist, whether accessing an incentive or avoiding an aversive (7), or with intrusions into personal space and territory (8). Frustration has been linked to displays of aggressive behavior to varying degrees, including redirected aggressive behavior (8–10). Frustration has been implicated in the performance of displacement behaviors (11) and repetitive behaviors including stereotypies (12). It is suggested that frustration evolved to invigorate responses when an individual is faced with threats to obtaining, protecting, and maintaining resources, and it is considered a negative emotional state, therefore frustration related behaviors are considered a potential welfare concern in animals (13, 14).

Like other affective states, frustration exists in the form of a specific emotional reaction, mood (period of irritability), and as a temperament trait (consistent behavioral predisposition over time and location). These forms have been investigated in the human literature: e.g., state vs. trait anger (encompassing some aspects of frustration) (15, 16) and a scale developed by Harrington (17) for measuring trait level frustration tolerance/intolerance (i.e., frustration tendencies). In dogs, like most other animal species, the focus of any research relating to frustration has tended to be on the immediate emotional reaction rather than the predisposition related to mood or temperament. For example, the frustration behaviors arising in domestic dogs when reinforcement for gazing at a human experimenter is extinguished included significant increases in frequency of ambulation, sniffing, and vocalizations (13). Other studies have explored changes in communicative aspects of dog-human behavior during reinforcement omission and extinction protocols (18, 19). Whilst such studies provide a foundation for how frustration may manifest in specific experimental settings, it is also important to understand the breadth of circumstances in which frustration can arise. In particular, considering frustration in the daily lives of owned dogs, and the reliability of a response across contexts, which may provide insight into a more general predisposition rather than a context specific response. In the field of clinical animal behavior, when assessing a problem, it is vital to understand both the motivation and the likely underlying emotion (e.g., differentiating fear/anxiety from frustration) so that specific treatment can be instituted (20). In addition, differentiating an individual dog who shows frustration in a single situation which is problematic (state level) from a dog who is generally frustration intolerant (trait level) is important, as they may require a different treatment approach.

In the daily lives of pet dogs, situations which may elicit frustration include the presence of physical barriers such as doors, or being restrained on a lead, both of which may thwart a dog from obtaining a desired resource (7). A desired resource may be a social (person or conspecific) or non-social (chasing prey, accessing food, a toy etc.) stimulus. In addition, frustration may arise alongside fear when access to safety is thwarted (21). Frustration may also arise in situations where expectations are not met due to absent, reduced or delayed reward (4). *Absence of a reward* may occur when an owner fails to provide access to a desired resource the dog was expecting (e.g., an owner may be in a rush to return from a walk and not allow the dog off lead to play with a conspecific as usual). *Reduced reward* occurs when a dog receives less than they were expecting while deviations from a set routine may result in frustration from a *delayed reward* (e.g., if a dog is walked or fed at a later time than usual). Situations where there is competition for a limited resource (e.g., one bone and two dogs who wish to have it; the shoe that the dog wants to chew but the owner wants back) can also result in frustration as there is a threat of loss of resources. Territory and personal space are also important resources (8) associated with increased autonomy—and so if a dog perceives a potential intrusion into his/her personal space and/or territory, frustration can arise. Indeed, a lack of autonomous control over the environment occurs in all of these contexts and is a contextual hallmark for frustration (22).

Given frustration exists to invigorate responses in order to increase focused efforts to achieve a desired goal, frustration related behaviors are likely to vary depending on the goal. However, typical component features (23) expected with frustration would include relatively high physiological arousal, communication of the desire for autonomy through aggressive displays (e.g., snarling, growling, snapping, biting) and behavioral tendencies associated with increased efforts such as pulling/lunging on lead or digging at a barrier to access the desired resource. Vocalizations (including whining, barking, growling) may accompany these efforts, and if the goal cannot be achieved then redirected behaviors (e.g., sudden grabbing of the lead) or displacement behaviors such as sniffing, scratching, spinning, or tail chasing may also be seen. Over time frustration may be implicated in the development of some repetitive and compulsive behaviors (24). It is unsurprising that given these responses, frustration is often implicated in many of the behavioral problems affecting dogs (7, 25). The form and intensity of frustration behavior can also result in a risk of injury to others. Despite the significance of these issues, the identification of individuals with poor frustration tolerance is currently based on the use of either instruments with facets loosely related to frustration [e.g., “self-assuredness” and “amicability” within the Monash Canine Personality Scale, (26)] or subjective evaluation by the clinician. The absence of a more precise and objective assessment instrument also impacts on the assessment of treatments aimed at controlling these problems, and is therefore a serious impediment to progress within the field. Therefore, the aim of the current study was to develop a psychometric instrument for the assessment of frustration in dogs via an owner completed questionnaire, encompassing common contexts, and manifestations of frustration in owned dogs.

## Materials and Methods

### Item Generation

A review of the literature on frustration in humans and non-human animals (including dogs) together with the data from three pieces of qualitative research was used to help generate items, namely:

1. The responses of semi-structured interviews with sixteen veterinary and non-veterinary behaviorists selected for their expertise and experience working with dogs in clinical animal behavior in order to provide face validity;
2. A request to all presenters from two consecutive years (2015–2016) of the International Society of Applied Ethology conference to provide their expert opinion on the expression of frustration in a species with which they work. Thirty-six respondents replied generating data with more comprehensive coverage on frustration relating to 14 species (including dogs);
3. A brief survey of dog owners on the University of Lincoln PetsCanDo database (http://www.lincolnpetscando.co.uk/) who were asked to comment on contexts and manifestations of frustration in their own dogs. Twenty-five dog owners replied providing data on 30 dogs to ensure the relevance of the phenomenon from a dog owner's point of view.

We sought to identify items encompassing the full range of aspects related to frustration in dogs identified from the literature review and qualitative research, for inclusion in the instrument. This included responses to frustration at the level of emotional reaction, mood and temperament, that considered its intensity, and where relevant, duration and frequency. Absent, reduced and delayed rewards that may trigger frustration were considered in both social and non-social contexts, including the typical barriers (e.g., lead restraint and confinement) that thwart an individual from achieving their goal. Aspects relating to lack of autonomous control, intrusion into territory/personal space, and the tendency to display repetitive behaviors were also included. Initially 33 items were included in the provisional list (please see Appendix 1 in Supplementary Material).

A 5-point agreement Likert scale was used for scoring items. Items were expressed as statements about the dog's behavior; an additional “Not applicable” (N/A) option was provided for each item to identify items that might be uncommon.

### Questionnaire Development

The questionnaire was written in British English. Demographic data was collected relating to the dog (breed, sex, neuter status, age, current and historic medical problems, current medication, data relating to training as well as behavior problems and treatments etc.). This was followed by the 33 items of the questionnaire relating to frustration. Five items were worded specifically for subsequent reverse scoring, in order to reduce the likelihood of a response set [these items are identified in Appendix 1 in Supplementary Material by **(R)**]. An additional question was included to determine concurrent validity with the construct of interest: “*I consider my dog to be very easily frustrated*.” Other items included asking owners about how well-behaved, and how obedient they felt their dog was, as well as how frequently they felt their dog experienced a potentially frustrating situation.

The questionnaire was tested for comprehensibility with 9 individual dog owners. Survey Monkey™ was used to develop an on-line version of the questionnaire. The questionnaire was circulated with the title: “*Profiling how your dog copes in a range of situations*,” avoiding the use of the word frustration so as to reduce the risk of bias. All respondents were asked to provide a contact e-mail address if they were willing to be contacted again about the study. This facilitated future reliability testing.

### Questionnaire Distribution

An electronic link to the questionnaire, including a brief summary of what it involved and an estimated time for completion, was distributed electronically using social media platforms [Facebook (https://www.facebook.com/) and Twitter (https://twitter.com/)] and via personal contacts of the authors. The stated inclusion criteria were (i) that the owner must be over 18 years old and (ii) that the questionnaire was completed about a dog the respondent currently owned. In order to take part in the survey, a participant was required to read the summary information then mark “agree” to proceed, constituting informed consent. The initial questionnaire was advertised and open for a period of 4 months from December 2016 to April 2017.

### Reliability Assessment

#### Intra-rater

In order to explore intra-rater reliability, respondents who provided an e-mail address to be contacted again were sorted by date of completion. Every second respondent was contacted a minimum of 6 weeks (maximum 7 weeks) after their first questionnaire completion, requesting they repeat the same questionnaire. The 6 week period was used to establish item reliability in the short term. The remaining half were contacted a minimum of 1 year (maximum 13 months) after completion of the initial questionnaire to establish temporal stability of the questionnaire. Between first and second completion, the order of the items/questionnaire layout was unchanged.

#### Inter-rater

All respondents who were contacted again were also asked to provide contact details if there was a second carer for their dog who would be willing to independently complete the same questionnaire about the same dog. The questionnaire was then sent to the second carer with the name of their dog, giving them the opportunity to complete the questionnaire.

### Statistical Analysis

All statistical analyses were conducted using IBM SPSS Version 22.

#### Item Scoring and Missing Data

The scoring method reflected the hypothesis that high scoring dogs should be experiencing greater frustration. A numerical score was given for each response from the 5-point Likert scale: 5 (“Strongly agree”), 4 (“Mainly agree”), 3 (“Partly agree, partly disagree”), 2 (“Mainly disagree”), or 1 (“Strongly disagree”). All items with anticipated “reverse” scoring requirements had their scores reversed before analysis (i.e., score of 5 becomes 1; 4 becomes 2; 3 remains 3; 2 becomes 4; 1 becomes 5). Complete datasets were those where there was a response for all 33 items (including “N/A” responses) therefore no imputation for missing data was required. “N/A” responses were handled in SPSS by “excluding pairwise”—i.e., the N/A response would be excluded, but the remaining item scores from that respondent would be included in the analysis. Incomplete questionnaires or those where a response set were identified were rejected from further analysis.

#### Intra- and Inter-rater Reliability

Non-parametric tests were used to assess correlation and significant differences to determine intra-rater reliability (at 6 weeks and 1 year) and inter-rater reliability of items. Items were excluded on the basis of significant differences (*p* < 0.05) between samples (Wilcoxon signed rank test), or non-significant (*p* > 0.05) and/or weak correlations (Spearman's rank order correlation < 0.2).

#### Content Structure

In order to maintain unidimensionality within the scale, but avoid redundancy from highly correlated items, the correlation matrix of all remaining items was inspected. Any items with no correlations >0.2 with another item, or with correlations >0.8 suggesting multicollinearity were removed. Items with anti-image matrices values >0.5 demonstrating sufficient sampling adequacy were retained (27).

Remaining items were then subject to principal component analysis (PCA) with a Varimax rotation (28). In order to decide how many components to extract, scree plots (29) alongside Kaiser's criterion (Eigenvalues >1) (30) were used. Items loading >0.4 were considered to significantly load on a given component (31). Cronbach's alpha (32) was used to measure internal consistency of the resulting scale.

#### Standardization of Overall Questionnaire Scores and Principal Component Scores

Standardization of scale scores was necessary to enable further comparison of dogs. Total scores were converted to a decimal for each dog, with “N/A” responses removed from the calculation. This generated a single “overall questionnaire score” (OQS) for each dog, with a range of possible scores from 0.2 to 1.0 [where OQS = s (total score achieved divided by total maximum score possible from the questions answered)]. The same process was also conducted to generate a score for each principal component for each dog.

#### Correlations With OQS/Principal Component Scores and Theoretically Related Concepts

Spearman's rank order correlations between the OQS/principal component scores and theoretically related concepts were explored to assess face validity. Concurrent validity was established from correlations and associated with owner rating on the specific item relating to how easily they believe their dog becomes frustrated “*I consider my dog to be very easily frustrated*.” Correlations between OQS/principal component scores and owner reported obedience, well-behaved-ness and the frequency with which their dog was exposed to frustrating situations were also explored.

#### Relationship Between OQS and Demographics/Dog Behavior History

Associations between the OQS and demographics/dog behavior history were analyzed using a general linear model (GLM), with OQS as the dependent variable. Explanatory factors included age as a covariate, and all categorical variables as fixed factors [sex; neuter status; breed; country of origin; size/bodyweight; place dog acquired; medical problem (current V none); presence/absence of owner reported behavior problem; whether the owner had sought help via a behavior consultation or not].

## Results

### Responses

Two thousand nine hundred and eighty-nine questionnaires were started on-line, and of these two thousand three hundred and forty-eight respondents completed the questionnaire to the end. Of these completed questionnaires, there were no missing data and there were no datasets where a response set was identified. The rate of “N/A” answers varied from 6 “N/A” responses (0.26%) for item 24 to 299 “N/A” responses (12.7%) for item 15. All items were therefore applicable to at least 87.3% of respondents, which was deemed to be acceptable at this stage.

Within the set of total respondents with completed questionnaires, 1,180 (50.3%) provided a name and e-mail address to be contacted again in the future.

### Demographics

Respondents were from 36 countries with: 1,365 (58.1%) from the United Kingdom; 703 (29.9%) from the USA. Respondents from all other countries were classes as “Other countries” for the purpose of analysis, including Canada [74 (3.2%)], Australia [48 (2.0%)], and a further 158 (6.8%) from 32 countries, with 18 (0.8%) respondents who did not provide a country.

The dataset included dogs aged from 2 months to 18 years 6 months old (average 5 years 11 months). The majority of dogs were neutered: male neutered (*n* = 938, 39.9%), female neutered (*n* = 905, 38.5%), male entire (*n* = 281, 12.0%), female entire (*n* = 208, 8.9%). Sixteen (0.7%) respondents did not provide their dog's sex/neuter status.

Dogs were assigned to 5 categories based on size: toy, < 5 kg (*n* = 81, 3.5%); small, 5–10 kg (*n* = 408, 17.4%); medium, 10–25 kg (*n* = 1,109, 47.2%); large, 25–45 kg (*n* = 674, 28.7%); giant, >45 kg (*n* = 74, 3.2%). Two (<0.1%) respondents did not provide their dog's size.

The majority of dogs were classed as pure bred (*n* = 1,489, 63.4%), with 674 cross bred dogs (28.7%). One hundred and twenty seven breeds were represented. For the purpose of analysis, breeds comprising >1% of the study population were considered as individual breeds (*n* = 11) with those representing <1% (*n* = 116) categorized as “Other pure bred” (see Appendix 6 in Supplementary Material). There were 185 respondents (7.9%) who did not select cross bred or a pure breed from the provided list−5 of these provided no breed related details, whilst the remaining 180 provided further details as to the specific breed(s) they classified their dog as (e.g., Labradoodle, Lurcher).

Overall, the majority of dogs were acquired from a “breeder” (*n* = 1,085, 46.2%), or a “shelter/ rescue” (*n* = 711, 30.3%). Other less common sources were classified as “Other source” for analysis (see Appendix 6 in Supplementary Material) and included: “neighbor/friend/relative” (*n* = 234, 10.0%), “adopted as a stray” (*n* = 66, 2.8%), “bred by myself” (*n* = 63, 2.7%), “pet store” (*n* = 21, 0.9%), and “other” (*n* = 164, 7.0%) where further details were provided (e.g., on-line advert, private rehome etc.).

### Distribution of Item Response Scores

Eight of the 33 items (items 9, 11, 12, 13, 17, 23, 31, 33) were grossly positively skewed from a normal distribution (i.e., mean <2 and median 1, all with Skewness values >1), but no items were grossly negatively skewed (i.e., mean >4 and median 5).

Using data for the 33 items, overall questionnaire scores (OQS) were calculated for each dog and visual inspection of the histogram appeared normally distributed, with a mean of 0.47 (±0.11) median 0.47, and a small positive skew (Skewness value 0.391).

#### Intra-rater/Test-Retest Responses

Spearman's rank order correlation and Wilcoxon signed rank test were conducted on all 33 items for intra-rater reliability assessment at 6 weeks and 1 year, and also for inter-rater reliability assessment as shown in Appendix 2 in Supplementary Material. The number of paired respondents is shown per item at each stage. A “N/A” rating resulted in excluding from analysis that item from that respondent.

Five hundred and ninety (590) respondents were contacted for the 6 week follow up. Of those contacted, 273 (46.3%) respondents completed the second questionnaire. Of these, 27 could not be paired with their original responses (*n* = 1 did not provide the dog's name; *n* = 26 provided a dog's name which could not be matched to an original questionnaire—it is suspected these owners erroneously completed the questionnaire for a different dog), leaving 246 completed paired questionnaires.

All items had a significant correlation between test and retest scores (*p* < 0.01). There were strong correlations (*r* = 0.5–1.0) for 27 items and moderate correlations (*r* = 0.3–49) for 6 items. Wilcoxon signed rank tests revealed a significant difference in the median test-retest scores of only one item (item 8, *r* = 0.024).

The remaining 590 respondents who provided an e-mail address and who had not been contacted at 6 weeks were contacted for the 1 year follow up. Of those contacted, 294 (49.8%) respondents completed the second questionnaire. Of these, 18 completed questionnaires could not be matched based on the details provided, leaving 276 completed paired questionnaires for 1 year intra-rater analysis.

All the items had a significant correlation between test and retest scores (*p* < 0.01). There were strong correlations (*r* = 0.5–1.0) for 18 items; moderate correlations (*r* = 0.3–0.49) for 14 items; and weak correlations (*r* = 0.1–0.29) for one item (item 15, *r* = 0.143). Wilcoxon signed rank tests revealed significant differences in the median test-retest scores for 4 items: items 4, 5, 28, and 31.

When age was explored, and dogs < 2 years old at completion of the first questionnaire (*n* = 33) were excluded, the analysis was rerun and item 4 could be retained (*n* = 243; *r* = 0.602, *p* < 0.01; Wilcoxon 0.110). There was no alteration in any other items to be included/excluded.

Given the increased number of items which were not reliable at 1 year, statistical analysis was rerun on the 276 paired responses, excluding those dogs where in the 1 year since owner completion of the first questionnaire there had been a change in any of the following categories: neutered (*n* = 5); developed new medical problems (*n* = 40); receiving new psychoactive medication (*n* = 14). Excluding these dogs did not alter the items to be excluded.

#### Inter-rater

One hundred and forty-eight respondents completed questionnaires for inter-rater reliability assessment. Of these, 56 could not be matched based on the details provided, leaving 92 completed paired questionnaires.

All items had a significant correlation between test and retest scores (*p* < 0.01) except for item 15 (*p* = 0.166) and item 17 (*p* = 0.352). There were strong correlations (*r* = 0.5–1.0) for 9 items; moderate correlations (*r* = 0.3–0.49) for 19 items; and weak correlations for 5 items (*r* = 0.1–0.29). Of the weak, the lowest correlations (<0.2) were for item 15 (*r* = 0.161) and item 17 (*r* = 0.100). Wilcoxon signed rank tests revealed significant differences in the median test-retest scores for 3 items: item 3 (0.040); item 20 (0.041); and item 24 (0.021).

Based on the above analysis 9 items were removed (items 3, 5, 8, 15, 17, 20, 24, 28, 31). Item 4 “*My dog is protective of his/her territory (house/garden/car),”* was retained after closer inspection as although it was not reliable at 1 year intra-rater reliability testing using all respondents' data, it was reliable when dogs who were <2 years old at the time of the first questionnaire being completed were excluded. This resulted in 24 items being taken forward for further analysis.

### Principal Component Analysis

The PCA was conducted on the 24 reliable items using the dataset of 2,348 respondents who had completed the original questionnaire. “N/A” answers were dealt with by excluding them pairwise.

Only item 7 had no correlations >0.2 with any other variables. There were no variables with correlations >0.8 (highest 0.585 between items 19 and 29) suggesting no problems with multicollinearity of data, and no singularity. Anti-image matrices showed that all items had the recommended value of >0.5 for sampling adequacy, except for item 27 which had a value of 0.478.

Based on this initial analysis, item 7 (“*My dog tends to react in the same way regardless of what he/she is frustrated by”*), and item 27 (“*My dog finds it very difficult to calm down if he/she does not get something they want”*) were removed.

PCA was run on the remaining 22 items with a Varimax orthogonal rotation.

Item 9 (“*My dog can lunge and grab at a nearby object* (e.g., *lead, clothing, toy, bed etc*.) *if he/she cannot access something wanted”*) did not load >0.4 on any component, so this was removed, and the analysis rerun resulting in all items loading >0.4 on at least one component (see Appendix 3 in Supplementary Material).

The final PCA had 5 components which explained 49.43% of the variance, converged in 9 iterations. The determinant was 0.009 (greater than the necessary 0.00001) (27). The Kaiser-Meyer-Olkin Measure of Sampling Adequacy KMO = 0.907 (i.e., “excellent”) (33) indicating confidence in the sample size being adequate for this analysis. In addition, Bartlett's test of Sphericity was highly significant χ^2^ (210) = 9628.310 (*p* < 0.001) (27).

Cross-loading >0.4 occurred only with item 25 *[“My dog gets upset if shut away from visitors (e.g., vocalizes or scratches/digs at the door)”];* loading on both component 2 (0.465) and component 4 (0.416). Given the higher relative loading, item 25 was considered as part of component 2.

### Biological Interpretation of Components

The items within each of the 5 components of the Varimax solution were deemed to be related and were labeled: PC1 (5 items) “*General frustration”*; PC 2 (4 items) “*Barrier frustration/perseverance”*; PC3 (4 items) “*Unmet expectations”*; PC4 (4 items) “*Autonomous control”*; and PC5 (3 items) “*Frustration coping”* (Table 1).

**Table 1:** Biological interpretation of principal components and variance explained by each −21 item, 5 component solution with Varimax rotation.

**PC**	**Item**	**Item**	**Name**	**Variance explained (%)**
PC1	19	My dog becomes frustrated in a large range of situations	General frustration	26.98
PC1	22	There are days when my dog seems to become more easily frustrated than others for no apparent reason		
PC1	29	My dog appears to become frustrated frequently (e.g., at least once daily)		
PC1	21	My dog shows increases in certain behaviors (e.g., lip licking, yawning, mounting, full body shake off) if he/she cannot immediately access something they want		
PC1	11	My dog engages in a repetitive behavior (e.g., tail chasing, pacing, circling) when unable to access something he/she wants		
PC2	26	My dog shows continued efforts (e.g., lunging, pulling toward) to approach a dog/person they wish to greet, when being restrained from doing so (e.g., when on lead)	Barrier frustration/perseverance	6.55
PC2	6	When on lead my dog will persist in lunging/pulling toward something he/she would like to chase (e.g., a cat, rabbit, bird, toy)		
PC2	18	My dog has difficulty in responding to cues/commands (e.g., sit, lie down, stay) if there is something else he/she wants to do or access		
PC2	25	My dog gets upset if shut away from visitors (e.g., vocalizes or scratches/digs at the door)		
PC3	2	My dog does not like being left out of activities with other dogs	Unmet expectations	5.70
PC3	32	My dog appears agitated and unsettled when he/she wants something another dog has (e.g., a toy or food item)		
PC3	30	My dog becomes very excited/restless (e.g., pacing, whining, barking, jumping up) when waiting to take part in an enjoyable activity		
PC3	1	My dog appears unsettled when there are delays in his/her routine (e.g., if walked or fed later than usual)		
PC4	13	My dog becomes aggressive (i.e., growl, snap, or bite) if I try to remove an item he/she has (e.g., favorite toy or food)	Autonomous control	5.25
PC4	23	When my dog is not kept busy, he/she can repeatedly lick, chew, or nibble their own body parts (e.g., paws, flanks/sides)		
PC4	33	My dog appears annoyed/upset if given less than he/she was expecting (e.g., wants table scrap and gets a pat on the head; given less food/a lower quality of food than expecting)		
PC4	12	My dog will attempt to escape if I try to confine him/her (e.g., in a room, crate, or kennel)		
PC4	4	My dog is protective of his/her territory (house, garden, car)		
PC5	14	My dog appears to cope well when denied access to things he/she is occasionally allowed (e.g., access to the sofa/bed or provision of table scraps) **(R)**	Frustration coping	5.02
PC5	16	I find it easy to interrupt/distract my dog from doing things he/she wants to do **(R)**		
PC5	10	My dog finds it easy to relax and settle when unable to access something he/she wants **(R)**		

In PC5, all 3 items (items 10, 14, and 16) require reverse scoring **(R)**, so once reverse scored, a high score represents low “Frustration coping” (i.e., a higher level of frustration intolerance) in-line with the scoring of other PCs.

### Internal Consistency

Overall Cronbach's alpha = 0.792, and evaluation of the effect of systematic deletion of components did not result in an increase in overall Cronbach's alpha (Appendix 4 in Supplementary Material) supporting the retention of all components. All PCs had a positive correlation with the OQS ranging from 0.596 for PC5 to 0.801 for PC1, supporting the hypothesis that such underlying components are related, but also reasonably separate (Appendix 5 in Supplementary Material).

### Standardization of Overall Questionnaire and Principal Component Scores

Principal component scores were calculated and OQS recalculated for the 21 items retained in the final solution for each dog, again accounting for any “N/A” answers, to standardize decimal scores between dogs (as shown in Table 2). Two response sets were identified, where an owner had answered “N/A” to all 21 retained items, therefore these were removed from analysis, leaving 2,346 respondents.

**Table 2:** Mean, standard deviation, median and skewness for 5 component, 21 item Varimax solution.

**Component**	**Mean**	**Standard deviation**	**Median**	**Skewness**
PC1	0.38	±0.15	0.36	0.919
PC2	0.55	±0.18	0.55	0.063
PC3	0.52	±0.17	0.50	0.186
PC4	0.37	±0.13	0.36	0.791
PC5	0.46	±0.16	0.47	0.535
OQS 21 item	0.45	±0.12	0.44	0.347

### Correlations With OQS/Principal Component Scores and Theoretically Related Concepts

A significant positive correlation was identified between the owner view of how easily frustrated they considered their dog to be (“*I consider my dog to be very easily frustrated*”) and the OQS and all principal components. This was strongest for PC1 (Spearman's *r* = 0.680) and OQS (Spearman's *r* = 0.646), with moderate correlations with other principal components (PC2: *r* = 0.408 PC3: *r* = 0.479; PC4: *r* = 0.399; PC5: *r* = 0.433). Weak to moderate negative correlations were identified between owner reported obedience levels [“*I consider my dog to be very obedient (i.e., is well trained and will respond to things I ask)”*] and OQS/all PCs. Stronger negative correlations were identified when looking at owner reported well-behaved-ness [“*I consider my dog to be very well-behaved (i.e., is able to manage his/her own behavior appropriately, without being told what to do*)”] and OQS/all PCs. Weak to moderate positive correlations were identified between the owner reported frequency with which their dog is exposed to frustrating situations (“*How often do you consider your dog to be exposed to a potentially frustrating situation?”*) and OQS/all PCs. Full results are shown in Appendix 5 in Supplementary Material.

### Relationship Between OQS and Demographics/Dog Behavior History

The full results of the GLM are shown in Table 3. Equal variances were assumed based on Levene's test (*p* = 0.795). It was established that a weak but significant negative correlation was found between age of dog when owner completed the questionnaire and OQS (Spearman's *r* = −0.148), where younger dogs to score more highly than older dogs. Therefore, in the GLM, “Age” was included as a covariate. Estimated marginal means (EMM) (taking into account “Age” as a covariate) for all dependent variables are shown in Appendix 6 in Supplementary Material.

**Table 3:** General linear model—“Overall questionnaire score” as dependent variable (significant effects at the level of *p* < 0.05 are highlighted in bold).

**Source**	**df**	***F***	***p***	**Partial Eta^**2**^**
Corrected Model	31	11.788	0.000	0.136
Intercept	1	114.385	**0.000**	0.047
Age (months)	1	48.469	**0.000**	0.021
Sex	2	4.757	**0.009**	0.004
Neuter status	2	5.290	**0.005**	0.005
Breed	12	1.784	**0.045**	0.009
Size	5	4.815	**0.000**	0.010
Source	3	0.994	0.394	0.001
Medical problem (current)	2	1.932	0.145	0.002
Behavior problem reported by owner	1	150.719	**0.000**	0.061
Behavior consult attended by owner	1	25.990	**0.000**	0.011
Country	2	1.692	0.184	0.001
Error	2,314			
Total	2,346			
Corrected Total	2,345			

*R Squared = 0.136 (Adjusted R Squared = 0.125)*.

Of the fixed factors, there was a significant effect of sex and neuter status, with male dogs scoring more highly than females, and neutered scoring more highly than unneutered. There was a significant effect of size on OQS, where increasing OQS was associated with decreasing size of dog. When considering breed, the 11 most common breeds from the study population were compared with “Cross-bred” and “Other pure-bred” dogs. The GLM suggested a marginal significant effect of breed (*p* = 0.045). However, *post-hoc* analysis based on EMM with Bonferroni adjustments for multiple comparisons revealed no significant differences between breeds.

The OQS of dogs with a behavior problem as reported by their owner was significantly higher than those with no reported problem. Similarly, the OQS of dogs who had attended a behavior consultation was significantly higher than those who had not.

When considering the “Source” of the dog, no significant difference was found between the categories “Breeder,” “Shelter” or “Other.” There was no significant difference in OQS of those dogs who had a current medical problem and those without. The country where the owner/dog was based had no significant effect on the OQS when the UK, USA and “Other” countries were compared.

## Discussion

The main aim of this study was to develop a reliable owner-based psychometric instrument for the assessment of frustration in dogs—the Canine Frustration Questionnaire (CFQ)—and we have succeeded in developing the first specific instrument of this kind. The importance of reliability as a core quality metric of a psychometric tool (34) was considered from the outset, hence reliability was established rigorously, at multiple levels in the development of this instrument. Whilst some other scales such as the Dog Impulsivity Assessment Scale (DIAS) have demonstrated long term temporal stability/reliability (35, 36), many developed scales fail to report reliability over time (37). The robustness of the specific items was facilitated by retaining only items shown to be reliable by the same raters after 6 weeks and by different raters familiar with the dog. The relatively high level of completion was probably in part a consequence of the initial qualitative research undertaken to identify relevant items followed by the comprehension analysis to ensure they were clearly understood. The soundness of our approach was supported by the results of the 1 year follow up which indicates that the latent traits underpinning responses are stable over time, as should be the case with features of personality/temperament.

The use of rigorous reliability assessment in scale development can come with a cost of removal of items important to the construct being measured, and potential loss of sensitivity. Only 9 of the original 33 items were lost during reliability assessment. Only 1 item was lost at 6 week intra-reliability assessment; and 3 further items were lost at 1 year intra-rater reliability assessment. The inter-rater reliability assessment is the single stage where most (*n* = 5) items were lost. This may not reflect problems with the facets of frustration that they assess, but rather different owners differing in their knowledge and perception of their dog. For example, the loss of item 17, “*My dog shows marked physical signs (e.g., panting, drooling, trembling) when he/she cannot access something they want”—*physical signs may not be uniformly recognized by all owners based on their level of experience. However, there may also be some differences in how a dog reacts depending on which owner/carer is present, and this may be particularly relevant in consideration of the loss of item 20 “*My dog will seek attention (e.g., looking at me, vocalizing, pawing, looking between me and the thing they want) when he/she wants something*.” A dog may learn that a given reaction may result in a particular outcome from one but not both owners (38)—this could be related to attention seeking, or could be related to differences between if/when an owner intervenes in such a context. One limitation from the process is that the final scale does not contain an item relating to intrusion into personal space; the relevant item (31) was removed at the 1 year intra-rater reliability assessment. However, further analysis reveals this item most highly correlates with *PC4—“Autonomous control”* (see Appendix 5 in Supplementary Material), therefore PC4 may be predictive of the response related to this item.

Item 4 *[“My dog is protective of his/her territory (house/garden/car)”]* was retained in the final questionnaire after reliability was demonstrated when excluding dogs who were <2 years old at the time of the first questionnaire being completed, i.e., immature dogs were excluded. Territoriality is deemed an important part of the construct of frustration and within the final structure appeared related to other items within *PC4—“Autonomous control.”* Our decision to raise the age of dogs for eligibility to consider this item is supported by the finding that the onset of aggressive behavior toward strangers is typically around 1.5 years (±0.2 years) and toward owners is 1.3 years (±0.1 years) (39). This suggests that this aspect of the trait may be subject to variability as the animal matures, and so is of clinical relevance. This is further supported by findings by Bamberger and Houpt (40) who found that the median age at which owners tend to seek help for behavior problems in their dogs (including “territorial aggression”) was 3.7 years (mean 2.5 years). As dogs age and reach sexual and social maturity, certain behavior changes can be seen and it is possible that “territoriality” does not truly develop and stabilize until social maturity is reached. It is important to consider that this single item may not be stable when assessing dogs <3 years old.

Content related validity was apparent in the form of both face and concurrent validity (34). Face validity comes from both the initial selection of items and the final structure of the instrument. In developing the instrument, as well as reviewing current literature on the topic, we sought advice from a wide range of experts in applied aspects of dog behavior and also animal welfare scientists working with dogs and other species, so as to provide broad coverage of items. In addition, initial stages also included consulting experienced owners on the contexts and manifestations of frustration in their dogs, so that the items included in the survey were likely translatable to the experiences of the dog owners. Concurrent validity was shown in relation to a number of expected correlates: the significant positive correlation between the OQS and owner view of how easily frustrated they considered their dog to be; the relationship between the presence of a behavior problem in a dog and the OQS; and, the likelihood that an owner had sought help via a behavior consultation. Such relationships are also consistent with the professional clinical observations of the authors, including two who are veterinary behavior specialists (HZ and DM).

Purported frustration levels from OQS/all PCs were negatively correlated with owner rating of well-behaved-ness and also, to a lesser extent obedience. The questionnaire qualified the concept of “well-behaved” for owners with an explanatory statement “*i.e., is able to manage his/her own behavior appropriately, without being told what to do*,” and also “obedience,” “*i.e., is well trained and will respond to things I ask*.” It is understandable that dogs scoring highly on the frustration scale would be less likely to be judged by their owners as being “well-behaved,” as it is likely that such dogs would struggle to manage their own behavior in various situations within everyday life, particularly when they are unable to achieve a particular goal. Whilst owner reported levels of “obedience” was also negatively correlated with the OQS, the correlation was, as expected, weaker. Considering obedience as responsiveness to cues, a dog may still respond to previously learned cues given by owners despite experiencing frustration, although it would be expected that when highly frustrated, a dog would respond less reliably to such cues i.e., an owner would be less likely able to distract, interrupt or redirect their dog at such times. This distinction is important to consider when devising programmes to help prevent or treat frustration related problems. Exercises such as those suggested by Zulch and Mills (41) to develop frustration tolerance (which do not focus on obedience), may therefore be important in such programmes, but have yet to be evaluated empirically. The development of the CFQ allows for the evaluation of such interventions in the future.

Convergent validity is provided by the positive correlations between OQSs and all PCs (Appendix 5 in Supplementary Material) as would be expected in a scale where all items and components are measuring aspects of a single trait. However, the PCs also indicate discriminant validity between separate facets related to frustration. The PCA explained about half of the variance in the dataset and so it must be appreciated that the precision within the scale may be relatively low due to other factors affecting the rating of the response of dogs in the contexts described. This is not surprising given the diversity of situations included in the rating which can induce frustration and the diversity of behaviors which dogs can potentially show given the options available in the environment at any given time. Nonetheless the first factor explained a substantial amount of the variance alone (27%) indicating that it provides a good guide to the frustration tendencies. All other items (which refer to more narrowly defined contexts) explained >5% (with Eigen values >1) and so are better on average than any single item within the questionnaire. These findings should also emphasize that scales such as this should not be used to define a discrete behavioral “disorder,” but rather as a guide to inform the assessor on an individual's predisposition toward different types of frustrative arousal.

In the following section we speculate about the clinical relevance of the different facets. Our biological interpretation of each PC facilitates predictions concerning the behavior and management of dogs scoring more highly within one domain. These should be considered hypotheses for consideration that can be tested through clinical intervention, and future work should aim to provide more empirical evidence in relation to these proposals:

(i) Dogs scoring highly on PC1—“*General frustration*” would be predicted to be experiencing and displaying signs of frustration regularly and in various aspects of their daily life. Interventions which focus on the development of general frustration tolerance may be particularly valuable (41). This component also contains the one item related to moodiness (item 22—“*There are days when my dog seems to become more easily frustrated than others for no apparent reason”)*, therefore PC1 might also include dogs with irritability and moodiness associated with pain (42). Whilst there was no significant difference in OQS from dogs with/without known current medical problems, it must be recognized that many chronic painful conditions manifesting as behavioral problems go unrecognized (42, 43);

(ii) Dogs scoring highly on PC2—“*Barrier frustration/perseverance*” would be predicted to persevere in attempting to achieve a specific goal, despite the presence of a physical barrier and at such a time, an owner may find them difficult to distract/interrupt. Interventions focused on building tolerance around a gradient of barriers may be useful in this instance;

(iii) Dogs scoring highly on PC3—“*Unmet expectations*” would be predicted to struggle to cope in situations where an expectation is not met, such as a routine change (absent or delayed reward). We would suggest that interventions focused on creating positive associations with change may be valuable (41);

(iv) Dogs scoring highly on PC4—“*Autonomous contro*l” might be expected to display problems (which may include aggressive behaviors) where there is a loss of freedom to act independently (due to restraint or confinement) and when there are threats to resources such as territory, food, or toys. Interventions based on teaching dogs to form positive associations with restraint, handling, confinement, and approach when in possession of resources may be useful here;

(v) Dogs scoring highly on PC5—“*Frustration coping*” would be predicted to struggle to relax and settle when faced with a situation where they cannot achieve their goal. Interventions which focus on teaching dogs how to cope with such disappointments may be most appropriate (41).

There are possible links between frustration tendencies measured by the CFQ (especially PC4—“*Autonomous control”*) and impulsivity, although this remains to be established. Impulsivity can be defined as a predisposition toward rapid, unplanned reactions without due consideration to the consequences (44). Whereas, impulsivity refers to the general executive control of behavior and cognition, frustration refers to a general emotional state. There is a substantial human literature to show that low frustration tolerance and impulsivity are often related within an individual, particularly in relation to aggressive behavior (45–48). More impulsive individuals may be more likely to place themselves in more frustrating situations due to poor decision making. This could be a result of either a failure to consider and thus anticipate the consequences of their action, or possible differences in sensitivity to reward or punishment. We therefore recommend that this scale be used and interpreted alongside those developed to measure impulsivity [DIAS, developed by Wright et al. (35)] and sensitivity to rewards and aversives [Positive and Negative Activation Scale, developed by Sheppard and Mills (49)]. The influence of impulsivity may also contribute to the relatively low level of variance explained by this scale.

Belief in low frustration tolerance has been linked to stress, anxiety, and depression in children (50). In addition, poor frustration tolerance has been suggested as a possible mechanism for the link between attention deficit hyperactivity disorder and co-morbid depression in children (51). It is possible that dogs may serve as a useful model for studying such human behavioral problems.

The finding that younger dogs tended to appear more highly frustration intolerant is in accordance with the finding that younger dogs have higher levels of impulsivity (35, 52) and positive activation (i.e., they have greater sensitivity to rewards) (49, 53). These two traits will make them more predisposed to frustration. Younger dogs are more interested in exploring and barriers to exploration such as being on lead, may result in higher levels of frustration. As they age, they may develop expectations based on what typically happens in a given situation and hence, frustration may reduce as expectations are realistic and largely met. Exceptions to this might relate to resources of value with respect to the individual's reproductive fitness, such as its territory, which may only become established as the animal reaches peak maturity, as discussed above.

When considering OQS and the effect of sex and neuter status, male dogs scored more highly than females and neutered dogs scored more highly than unneutered. Neutering may have an adverse impact on certain behavior problems as suggested in male dogs by McGreevy et al. (54), however it may be that more frustrated dogs are more likely to be neutered in order to help control the associated problems (55–57). It is also possible that entire/unneutered dogs typically develop more frustration tolerance as they have to cope routinely with denial of access to a sexual partner in our society. Alternatively, entire dogs may be managed more carefully in some contexts such as around other dogs, to avoid unwanted breeding or interactions with the opposite sex. It is difficult to draw any definite conclusions from these results, but the relationship deserves further investigation in a longitudinal study to elucidate causal relationships between neutering and frustration tolerance.

Whether dogs were acquired from a breeder, shelter, or other source made no significant difference to the OQS. Despite this, behavior problems are a common cause of dogs ending up in a shelter (58), including those likely related to frustration such as destructive and aggressive behavior (59), so these should still be considered as potential reasons for dogs being relinquished.

Another potentially surprising relationship is the weak to moderate positive correlation between the OQS and the reported frequency of exposure to frustrating situations. There are several possible reasons for this: a dog owner may find managing the overt behaviors of a dog experiencing more frustration challenging, and so may have a tendency to avoid situations which may trigger these responses meaning a reduced frequency of exposure; alternatively, a dog may start to habituate to frustrating situations if they experience them regularly. This again emphasizes the need to develop effective evidence-based interventions to help manage and potentially prevent this problem.

There was a trend where OQS decreased as size of dog increased, i.e., smaller dogs scored more highly on the CFQ OQS. This size related effect is consistent with aggressiveness toward people as reported by Martínez et al. (60) and prevalence of behavior problems reported by McGreevy et al. (61). Whilst it has been suggested that this may have a genetic basis (62), there are other explanations for this observation. It is possible that management plays a role, with smaller dogs possibly more likely to be lifted/carried or physically restrained compared to their larger counterparts, which may lead to increased frustration related issues related to autonomous control. Another possible explanation is that frustration related behaviors and associated risks may be better tolerated (and therefore not addressed) by owners of smaller dogs compared to owners of larger dogs, as found by Guy et al. (63).

Whilst the questionnaire was only distributed in English, owners who completed it were from a wide range of countries. The lack of a significant difference between OQS from dogs based in different countries reinforces our assertion that the psychometric tool is generally reliable and so applicable to owners and their dogs in countries other than the UK.

However, we concede that the instrument is based on expert opinion and owners' perception of frustration in dogs. In order to further validate the tool, correlations between the questionnaire and a suite of behavioral tests designed to replicate the biological associations proposed for each specific principal component needs to be undertaken Additionally, physiological correlates should be explored, which may provide further validation and may also guide future targeted pharmacological interventions for treating those dogs with frustration related problems. Exploring correlations between scores on the CFQ and other psychometric scales such as the DIAS and PANAS would be interesting to elucidate relationships between these temperament/personality scales. All of these tasks are currently being undertaken by the authors.

In conclusion, the CFQ can be considered a robust tool to measure frustration tendencies in dogs, which demonstrates much validity and reliability. The introduction of the CFQ into clinical behavior practice will enable not only the identification of dogs with frustration tendencies, but also the future evaluation of interventions to specifically manage such problems. Further validation of the tool with behavioral and physiological correlates is underway, to increase confidence in this previously neglected area.

## Data Availability

The datasets generated for this study are available on request to the corresponding author.

## Ethics Statement

This study was granted ethical approval by the College of Science Research Ethics Committee, University of Lincoln (reference CoSREC160).

## Author Contributions

The idea for the study was devised by KM, LC, HZ, and DM. KM distributed the questionnaire and collected responses. KM, LC, HZ, and DM all contributed to the analysis of the results and the creation of the final manuscript.

### Conflict of Interest Statement

The authors declare that the research was conducted in the absence of any commercial or financial relationships that could be construed as a potential conflict of interest.
