# Supplementary material for: The Canine Frustration Questionnaire—Development of a New Psychometric Tool for Measuring Frustration in Domestic Dogs (Canis familiaris)
Source: Front Vet Sci. 2019 May 17;6:152. doi: 10.3389/fvets.2019.00152 (PMC6535675; doi:10.3389/fvets.2019.00152)
Supplement: Supplementary file 1 [file Data_Sheet_1.docx]

| **Item no.** | **Item after comprehensibility** | **Reverse Scored (R)** |
| --- | --- | --- |
| 1 | My dog appears unsettled when there are delays in his/her routine (e.g. if walked or fed later than usual) |  |
| 2 | My dog does not like being left out of activities with other dogs |  |
| 3 | My dog gives up quickly (e.g. moving away from/avoiding) when he/she cannot access something wanted | **(R)** |
| 4 | My dog is protective of his/her territory (house, garden, car) |  |
| 5 | My dog becomes highly excited and agitated whilst waiting for his/her food |  |
| 6 | When on lead my dog will persist in lunging/pulling towards something he/she would like to chase (e.g. a cat, rabbit, bird, toy) |  |
| 7 | My dog tends to react in the same way regardless of what he/she is frustrated by |  |
| 8 | During periods when my dog does less than usual (e.g. reduced walks, less than expected food or sessions of play) he/she appears to become agitated/upset |  |
| 9 | My dog can lunge and grab at a nearby object (e.g. lead, clothing, toy, bed etc.) if he/she cannot access something wanted |  |
| 10 | My dog finds it easy to relax and settle when unable to access something he/she wants | **(R)** |
| 11 | My dog engages in a repetitive behaviour (e.g. tail chasing, pacing, circling) when unable to access something he/she wants |  |
| 12 | My dog will attempt to escape if I try to confine him/her (e.g. in a room, crate or kennel) |  |
| 13 | My dog becomes aggressive (i.e. growl, snap or bite) if I try to remove an item he/she has (e.g. favourite toy or food) |  |
| 14 | My dog appears to cope well when denied access to things he/she is occasionally allowed (e.g. access to the sofa/bed or provision of table scraps) | **(R)** |
| 15 | My dog tends to react in the same way when frustrated by the same thing on repeated occasions |  |
| 16 | I find it easy to interrupt/distract my dog from doing things he/she wants to do | **(R)** |
| 17 | My dog shows marked physical signs (e.g. panting, drooling, trembling) when he/she cannot access something they want |  |
| 18 | My dog has difficulty in responding to cues/commands (e.g. sit, lie down, stay) if there is something else he/she wants to do or access |  |
| 19 | My dog becomes frustrated in a large range of situations |  |
| 20 | My dog will seek attention (e.g. looking at me, vocalising, pawing, looking between me and the thing they want) when he/she wants something |  |
| 21 | My dog shows increases in certain behaviours (e.g. lip licking, yawning, mounting, full body shake off) if he/she cannot immediately access something they want |  |
| 22 | There are days when my dog seems to become more easily frustrated than others for no apparent reason |  |
| 23 | When my dog is not kept busy, he/she can repeatedly lick, chew or nibble their own body parts (e.g. paws, flanks/sides) |  |
| 24 | My dog is very patient when waiting for something he/she wants | **(R)** |
| 25 | My dog gets upset if shut away from visitors (e.g. vocalises or scratches/digs at the door) |  |
| 26 | My dog shows continued efforts (e.g. lunging, pulling towards) to approach a dog/person they wish to greet, when being restrained from doing so (e.g. when on lead) |  |
| 27 | My dog finds it very difficult to calm down if he/she does not get something they want |  |
| 28 | My dog shows increases in certain behaviours (e.g. barking, whining, growling, jumping up) if he/she cannot immediately access something they want |  |
| 29 | My dog appears to become frustrated frequently (e.g. at least once daily) |  |
| 30 | My dog becomes very excited/restless (e.g. pacing, whining, barking, jumping up) when waiting to take part in an enjoyable activity |  |
| 31 | My dog is intolerant of intrusion into his/her personal space (e.g. being approached when resting or being physically restrained) |  |
| 32 | My dog appears agitated and unsettled when he/she wants something another dog has (e.g. a toy or food item) |  |
| 33 | My dog appears annoyed/upset if given less than he/she was expecting (e.g. wants table scrap – gets a pat on the head; given less food/a lower quality of food than expecting) |  |

Appendix 1 - Thirty-three items used for frustration questionnaire after comprehensibility testing

| **Item no.** | **6 week intra-rater** | | | | **1 year intra-rater** | | | | **Inter rater** | | | |
| --- | --- | --- | --- | --- | --- | --- | --- | --- | --- | --- | --- | --- |
|  | **Spearman’s** | **p** | **n** | **Wilcoxon** | **Spearman’s** | **p** | **n** | **Wilcoxon** | **Spearman’s** | **p** | **n** | **Wilcoxon** |
| 1 | 0.620 | 0.000 | 242 | 0.801 | 0.526 | 0.000 | 264 | 0.085 | 0.535 | 0.000 | 88 | 0.061 |
| 2 | 0.713 | 0.000 | 213 | 0.530 | 0.587 | 0.000 | 249 | 0.501 | 0.351 | 0.002 | 76 | 0.649 |
| 3 | 0.449 | 0.000 | 243 | 0.075 | 0.417 | 0.000 | 264 | 0.506 | 0.314 | 0.003 | 87 | 0.040* |
| 4 | 0.692 | 0.000 | 244 | 0.057 | 0.611 | 0.000 | 272 | 0.012* | 0.383 | 0.000 | 90 | 0.299 |
| 5 | 0.711 | 0.000 | 241 | 0.241 | 0.660 | 0.000 | 272 | 0.001* | 0.550 | 0.000 | 91 | 0.236 |
| 6 | 0.677 | 0.000 | 241 | 0.548 | 0.610 | 0.000 | 273 | 0.331 | 0.555 | 0.000 | 88 | 0.417 |
| 7 | 0.500 | 0.000 | 229 | 0.082 | 0.309 | 0.000 | 242 | 0.349 | 0.304 | 0.005 | 84 | 0.399 |
| 8 | 0.564 | 0.000 | 239 | 0.024* | 0.536 | 0.000 | 268 | 0.275 | 0.387 | 0.000 | 91 | 0.969 |
| 9 | 0.557 | 0.000 | 233 | 0.380 | 0.541 | 0.000 | 263 | 0.990 | 0.411 | 0.000 | 91 | 0.818 |
| 10 | 0.465 | 0.000 | 241 | 0.695 | 0.500 | 0.000 | 275 | 0.791 | 0.304 | 0.003 | 91 | 0.930 |
| 11 | 0.495 | 0.000 | 236 | 0.987 | 0.429 | 0.000 | 267 | 0.373 | 0.302 | 0.005 | 85 | 0.834 |
| 12 | 0.569 | 0.000 | 236 | 0.548 | 0.521 | 0.000 | 261 | 0.880 | 0.296 | 0.005 | 87 | 0.646 |
| 13 | 0.732 | 0.000 | 244 | 0.551 | 0.625 | 0.000 | 270 | 0.746 | 0.554 | 0.000 | 92 | 0.258 |
| 14 | 0.374 | 0.000 | 234 | 0.364 | 0.400 | 0.000 | 269 | 0.818 | 0.314 | 0.003 | 86 | 0.369 |
| 15 | 0.380 | 0.001 | 214 | 0.250 | 0.143* | 0.030 | 229 | 0.181 | 0.161* | 0.166* | 76 | 0.577 |
| 16 | 0.498 | 0.000 | 246 | 0.813 | 0.453 | 0.000 | 275 | 0.924 | 0.243 | 0.021 | 90 | 0.115 |
| 17 | 0.502 | 0.000 | 234 | 0.120 | 0.451 | 0.000 | 273 | 0.249 | 0.100* | 0.352* | 88 | 0.256 |
| 18 | 0.549 | 0.000 | 245 | 0.996 | 0.451 | 0.000 | 275 | 0.223 | 0.371 | 0.000 | 92 | 0.164 |
| 19 | 0.665 | 0.000 | 243 | 0.385 | 0.509 | 0.000 | 272 | 0.262 | 0.516 | 0.000 | 89 | 0.761 |
| 20 | 0.557 | 0.000 | 245 | 0.615 | 0.524 | 0.000 | 274 | 0.193 | 0.371 | 0.000 | 92 | 0.041* |
| 21 | 0.559 | 0.000 | 239 | 0.656 | 0.455 | 0.000 | 271 | 0.244 | 0.329 | 0.002 | 89 | 0.628 |
| 22 | 0.540 | 0.000 | 236 | 0.226 | 0.477 | 0.000 | 269 | 0.131 | 0.265 | 0.013 | 87 | 0.076 |
| 23 | 0.664 | 0.000 | 231 | 0.560 | 0.474 | 0.000 | 266 | 0.221 | 0.484 | 0.000 | 90 | 0.642 |
| 24 | 0.681 | 0.000 | 246 | 0.468 | 0.534 | 0.000 | 274 | 0.850 | 0.376 | 0.000 | 92 | 0.021* |
| 25 | 0.657 | 0.000 | 227 | 0.305 | 0.625 | 0.000 | 240 | 0.454 | 0.560 | 0.000 | 85 | 0.705 |
| 26 | 0.626 | 0.000 | 242 | 0.335 | 0.589 | 0.000 | 264 | 0.976 | 0.513 | 0.000 | 90 | 0.957 |
| 27 | 0.603 | 0.000 | 243 | 0.135 | 0.465 | 0.000 | 273 | 0.524 | 0.487 | 0.000 | 91 | 0.250 |
| 28 | 0.631 | 0.000 | 242 | 0.432 | 0.566 | 0.000 | 272 | 0.006* | 0.460 | 0.000 | 91 | 0.434 |
| 29 | 0.624 | 0.000 | 244 | 0.270 | 0.492 | 0.000 | 273 | 0.172 | 0.375 | 0.000 | 92 | 0.465 |
| 30 | 0.554 | 0.000 | 244 | 0.834 | 0.599 | 0.000 | 271 | 0.422 | 0.527 | 0.000 | 92 | 0.221 |
| 31 | 0.517 | 0.000 | 240 | 0.098 | 0.375 | 0.000 | 269 | 0.005* | 0.325 | 0.000 | 90 | 0.423 |
| 32 | 0.633 | 0.000 | 214 | 0.233 | 0.534 | 0.000 | 246 | 0.460 | 0.548 | 0.000 | 81 | 0.783 |
| 33 | 0.601 | 0.000 | 239 | 0.370 | 0.419 | 0.000 | 263 | 0.075 | 0.439 | 0.000 | 86 | 0.344 |

Appendix 2 - Intra-rater (6 week and 1 year) and inter-rater reliability analysis (*items removed based on Spearman’s correlation <0.2 and/or p<0.05, or Wilcoxon p<0.05)

| **Rotated Component Matrix** | | | | | |
| --- | --- | --- | --- | --- | --- |
|  | Component | | | | |
|  | PC1 | PC2 | PC3 | PC4 | PC5 |
| Q19 | **.704** | *.286* | *.131* | *.107* | *.133* |
| Q22 | **.692** | *.157* | *.169* | *.093* | *-.035* |
| Q29 | **.649** | *.172* | *.259* | *.119* | *.237* |
| Q21 | **.581** | *.107* | *.274* | *.192* | *.072* |
| Q11 | **.553** | *.026* | *.048* | *.170* | *.098* |
| Q26 | *.112* | **.788** | *.147* | *.112* | *.047* |
| Q6 | *.197* | **.751** | *.131* | *.064* | *-.021* |
| Q18 | *.346* | **.575** | *.029* | *.043* | *.227* |
| Q25 | *-.034* | **.465** | *.297* | **.416** | *.138* |
| Q2 | *.031* | *.217* | **.746** | *-.124* | *.059* |
| Q32 | *.260* | *.117* | **.549** | *.325* | *.029* |
| Q30 | *.203* | *.301* | **.545** | *.136* | *-.001* |
| Q1 | *.304* | *-.087* | **.487** | *.040* | *.134* |
| Q13 | *.145* | *.049* | *-.075* | **.623** | *.177* |
| Q23 | *.366* | *.050* | *.001* | **.503** | *-.040* |
| Q33 | *.275* | *-.006* | *.359* | **.473** | *.155* |
| Q12 | *-.032* | *.304* | *.108* | **.441** | *.301* |
| Q4 | *.206* | *.179* | *.167* | **.432** | *-.355* |
| Q14 | *.045* | *-.010* | *.112* | *.241* | **.690** |
| Q16 | *.335* | *.283* | *-.041* | *.085* | **.548** |
| Q10 | *.316* | *.153* | *.287* | *-.032* | **.512** |

Appendix 3- Structure matrix for final 5 principal component, 21 item Varimax solution (bold font = those items loading >0.4; grey cells = those items included in each principal component (PC))

| **Item-Total Statistics** | | | | | |
| --- | --- | --- | --- | --- | --- |
|  | **Scale Mean if Item Deleted** | **Scale Variance if Item Deleted** | **Corrected Item-Total Correlation** | **Squared Multiple Correlation** | **Cronbach's Alpha if Item Deleted** |
| **PC1** | 1.9096 | .232 | .653 | .434 | .727 |
| **PC2** | 1.7309 | .215 | .598 | .369 | .746 |
| **PC3** | 1.7619 | .225 | .594 | .367 | .745 |
| **PC4** | 1.9121 | .262 | .568 | .338 | .759 |
| **PC5** | 1.8219 | .252 | .475 | .238 | .782 |

Appendix 4 - Cronbach's alpha for overall score for each principal component

|  | **OQS** | **PC1** | **PC2** | **PC3** | **PC4** | **PC5** | **Owner reported ease at which frustrated** | **Owner reported obedience** | **Owner reported well-behavedness** | **Frequency exposed to frustrating situation** | **Item 31- intrusion into personal space** |
| --- | --- | --- | --- | --- | --- | --- | --- | --- | --- | --- | --- |
| **OQS** | 1.000 | .806^**^ | .772^**^ | .753^**^ | .717^**^ | .596^**^ | .646^**^ | -.394** | -.490** | .274** | .306** |
| **PC1** | .806^**^ | 1.000 | .495^**^ | .519^**^ | .502^**^ | .425^**^ | .680^**^ | -.303** | -.423** | .346** | .303** |
| **PC2** | .772^**^ | .495^**^ | 1.000 | .496^**^ | .456^**^ | .352^**^ | .408^**^ | -.399** | -.445** | .146** | .151** |
| **PC3** | .753^**^ | .519^**^ | .496^**^ | 1.000 | .419^**^ | .344^**^ | .479^**^ | -.194** | -.291** | .216** | .203** |
| **PC4** | .717^**^ | .502^**^ | .456^**^ | .419^**^ | 1.000 | .323^**^ | .399^**^ | -.261** | -.278** | .139** | .327** |
| **PC5** | .596^**^ | .425^**^ | .352^**^ | .344^**^ | .323^**^ | 1.000 | .433^**^ | -.331** | -.413** | .180** | .165** |
| **Owner reported ease at which frustrated** | .646^**^ | .680^**^ | .408^**^ | .479^**^ | .399^**^ | .433^**^ | 1.000 | -.264^**^ | -.426^**^ | .378^**^ | .287^**^ |
| **Owner reported obedience** | -.394^**^ | -.303^**^ | -.399^**^ | -.194^**^ | -.261^**^ | -.331^**^ | -.264^**^ | 1.000 | .595^**^ | -.070^**^ | -.096^**^ |
| **Owner reported well-behavedness** | -.490^**^ | -.423^**^ | -.445^**^ | -.291^**^ | -.278^**^ | -.413^**^ | -.426^**^ | .595^**^ | 1.000 | -.169^**^ | -.155^**^ |
| **Frequency exposed to frustrating situation** | .274^**^ | .346^**^ | .146^**^ | .216^**^ | .139^**^ | .180^**^ | .378^**^ | -.070^**^ | -.169^**^ | 1.000 | .103^**^ |
| **Item 31- intrusion into personal space** | .306^**^ | .303^**^ | .151^**^ | .203^**^ | .327^**^ | .165^**^ | .287^**^ | -.096^**^ | -.155^**^ | -.103^**^ | 1.000 |

Appendix 5 – Spearman’s rank order correlations between OQS, principal components and owner reported obedience, well-behavedness, frequency exposed to a frustrating situation and removed item 31 (intrusion into personal space) (all correlations significant at the p<0.001 level)

| **Fixed factor** | **Groups** | **Mean** | **Std. Error** | **95% Confidence Interval** | |
| --- | --- | --- | --- | --- | --- |
|  |  |  |  | **Lower Bound** | **Upper Bound** |
| Sex | Female | .417^a^ | .032 | .355 | .479 |
|  | Male | .431^a^ | .032 | .369 | .494 |
| Neuter status | No – not neutered | .375^a^ | .032 | .312 | .438 |
|  | Yes - neutered | .394^a^ | .032 | .331 | .457 |
| Size | Giant (>45kg/ >99lbs) | .389^a^ | .043 | .305 | .474 |
|  | Large (25-45kg/ 55-99lbs) | .406^a^ | .041 | .325 | .486 |
|  | Medium (10-25kg/ 22-55lbs) | .410^a^ | .041 | .329 | .490 |
|  | Small (5-10kg/ 11-22lbs) | .436^a^ | .041 | .355 | .517 |
|  | Toy (<5kg/<11lbs) | .440^a^ | .043 | .355 | .524 |
| Breed | CROSSBREED | .413^a^ | .041 | .332 | .494 |
|  | Australian Shepherd | .439^a^ | .046 | .350 | .528 |
|  | Beagle | .454^a^ | .047 | .362 | .546 |
|  | Border Collie | .403^a^ | .042 | .321 | .486 |
|  | Border Terrier | .413^a^ | .047 | .322 | .505 |
|  | Cocker Spaniel | .406^a^ | .043 | .322 | .490 |
|  | English Springer Spaniel | .390^a^ | .044 | .304 | .477 |
|  | German Shepherd | .411^a^ | .043 | .328 | .495 |
|  | Golden Retriever | .381^a^ | .044 | .295 | .466 |
|  | Jack Russell Terrier | .441^a^ | .044 | .355 | .528 |
|  | Labrador Retriever | .399^a^ | .042 | .316 | .482 |
|  | Staffordshire Bull Terrier | .420^a^ | .043 | .335 | .504 |
|  | OTHER PURE BRED | .401^a^ | .041 | .320 | .481 |
| Behaviour problem | No | .353^a^ | .042 | .271 | .435 |
|  | Yes | .474^a^ | .041 | .393 | .554 |
| Behaviour consult | No | .396^a^ | .041 | .316 | .477 |
|  | Yes | .430^a^ | .042 | .349 | .512 |
| Source | Breeder | .427^a^ | .039 | .349 | .504 |
|  | Shelter/rescue | .431^a^ | .040 | .353 | .508 |
|  | OTHER SOURCE | .421^a^ | .040 | .343 | .499 |
| Medical problem | No medical problem | .407^a^ | .041 | .327 | .487 |
|  | Yes – Current medical problem | .416^a^ | .041 | .337 | .496 |
| Country | UK | .413^a^ | .041 | .332 | .493 |
|  | USA | .420^a^ | .041 | .339 | .502 |
|  | OTHER COUNTRY | .407^a^ | .042 | .325 | .489 |
| a. Covariates appearing in the model are evaluated at the following values:  AgeMonths = 70.6957 | | | | | |

Appendix 6 – Estimated marginal means for all fixed factors
